# NEFA, BHBa, UREA and Liver Enzyme Variation in the Bloodstream of Weaned Foals up to 18 Months of Age

**DOI:** 10.3390/ani11061746

**Published:** 2021-06-11

**Authors:** Maria Grazia Cappai, Andrea Taras, Giovanni Paolo Biggio, Corrado Dimauro, Domenico Gatta, Ignazio Cossu, Raffaele Cherchi, Walter Pinna

**Affiliations:** 1 Dipartimento di Medicina Veterinaria, University of Sassari, 07100 Sassari, Italy; prodanim@uniss.it; 2 Dipartimento di Ricerca per gli Equini, Agris Sardegna, 07014 Ozieri, Italy; andreataras@yahoo.it (A.T.); giampaolo.biggio@tiscali.it (G.P.B.); icossu@agrisricerca.it (I.C.); ilex283@gmail.com (R.C.); 3 Dipartimento di Agraria, University of Sassari, 07100 Sassari, Italy; dimauro@uniss.it; 4 Dipartimento di Scienze Veterinarie, University of Pisa, 56124 Pisa, Italy; domenico.gatta@unipi.it

**Keywords:** body depots, energy balance, ketone bodies, liver, mass development

## Abstract

**Simple Summary:**

Energy balance assessment of the growing horse requires a complex nutritional evaluation. Some biochemical parameters can be of clinical importance to prevent the onset of metabolic disorders. The adequate nourishment of body tissues in the growing foal may represent an issue in the practice, given the dynamic change of body composition, in view of the potential mobilization of fat from tissue depots and increasing lean mass. As such, the maintenance of adequate body weight and body measures over time (optimal growth curve accomplishment) and the fulfillment of nutrient requirements are cornerstones of individual feeding plans for the expression of the athletic potential of the future sport horse. In this scenario, the metabolic evaluation of growing foals turned out to be a valuable tool to consider the energy distribution within the animal body. In view of those particular conditions, non-esterified fatty acids (NEFA), beta-hydroxybutyric acid (BHBa), UREA and liver enzymes showed to serve as indicators to monitor energy balance and health in growing foals from weaning to 18 months of age.

**Abstract:**

The pattern of selected metabolites for interpreting homeostasis during the growth of foals can be used as an indicator of energy balance state and liver health. Against this background, the literature on circulating parameters of foals across growth stages is scanty. We hypothesized that circulating metabolites indicating energy distribution such as non-esterified fatty acids (NEFA), β-hydroxy-butyric acid (BHBa), UREA and liver enzyme-like γ-glutamyl-transferase (γ-GT) [interpreted in the light of circulating total bilirubin (TBIL), alanine aminotransferase (ALT) and aspartate aminotransferase (AST)] may be used to monitor the energy balance of growing foals. A total of 12 Anglo-Arab (AA) foals from the same stable were enrolled in this trial. All foals were serially weighed on a digital scale and sampled for total blood at weaning, at 12 and 18 months of age. Feeding and keeping conditions were similar for all the foals involved. Animals appeared healthy and no signs of poor growth performance were pointed out. The peak of circulating NEFA mobilized from body depots was reached at one year of age but markedly dropped at 18 months, when BHBa increased (*p* < 0.001) alongside with liver enzyme. BHBa and γ-GT levels turned out to positively correlate (*p* = 0.051). However, at 6, 12 and 18 months, γ-GT dropped in the physiological reference range for the horse, thus showing no prognostic value. ALT and UREA significantly increased (*p* = 0.008 and *p* = 0.006, respectively) when NEFA also increased (*p* = 0.001). Liver enzyme increase could be associated with fat mobilization and ketone bodies production meanwhile amino acid transamination for energy purposes led to the increase of UREA in the bloodstream. However, no prognostic value to liver enzyme could be attributed in this trial.

## 1. Introduction

The growing foal shows different stages of body development over time [1,2,3,4,5]. The increase in mass and size occurs following different gains across distinct periods of growth so that curves could be developed, according to horse breed. In addition, the speed of growth, as well as the morphometric development (height at withers, for instance) and weight gains, may change according to a series of factors, namely birth period, maternal effects and management [6,7,8,9,10]. However, independent of differences in growth speed and performance of horse breeds, the daily energy requirement is augmented in the foal and serves to accomplish the high demand of the developing tissues. Therefore, the energy for harmonic growth and development of tissues and cognitive functions should be adequately assured for keeping foals healthy. 

The nutritional assessment is of clinical importance to prevent the onset of metabolic disorders from energy and nutrients imbalances. Recently, the evaluation of the nutritional state has been accounted as the fifth vital assessment in companion animals [11]. Following an integrated approach to the exploration of nourishment level of the different tissues composing the animal body [12], the metabolic profile appears to be a useful tool for monitoring the evolution of growth and energy distribution, thanks to the determination of circulating levels of selected biochemical parameters, playing as indicators of energy mobilization from fat depots and use of substrates when the energy balance is negative.

Thus, circulating levels of non-esterified fatty acids (NEFA) may turn to be useful to provide a measure of the lipomobilization from body storage [13,14]. However, when fat is intensively mobilized to provide energy at a systemic level, as in the case of augmented energy demand exceeding that obtained with the diet, also other ketone bodies can be synthesized and, as a consequence, their level in the bloodstream increases [14]. In particular, beta-hydroxybutyric acid (BHBa) is formed in the hepatocyte as an intermediate metabolite for energy purposes, serving as an alternative substrate for cell metabolism. However, in the condition of negative energy balance horses can turn to protein catabolism and increase significantly the gluconeogenic pathway from amino acids, with a consequent increase in ammonia production, converted to UREA for elimination [15]. 

The body condition scoring (BCS) system for the assessment of fatness in the horse [16] is of difficult application in the practice for the evaluation in the young foal during growth. This is mainly due to the continuous change of proportions between fat and lean masses and the ideal body weight for each growth stage is hard to define. In view of such practical hints, the appraisal of body condition ranging from underweight to overweight appears more feasible, though less accurate. 

Against this background, it was hypothesized that NEFA and BHBa, involved in the biochemical patterns for energy production at a systemic level, could serve as good indicators of energy balance and distribution over time, across different growth stages of the foal. Therefore, the present investigation aimed to monitor and interpret the shift of circulating values of NEFA, BHBa, UREA and liver enzymes with other selected metabolites at different growth stages of spring-born foals, from weaning to 18-months of age. Overall metabolic and health conditions were monitored, in view of the consequential phases of feeding and management practices. 

## 2. Materials and Methods

### 2.1. Animal Care

Animal handling complied with the recommendations of European Union Directive 2010/63/EU concerning animal care. All procedures reported in this trial belong to conventional clinical practices; in particular, blood sampling and body measure collection were carried out by expert veterinary practitioners and trained technicians. This article complies with the recommendations for the conduct, reporting and publication of scholarly work in medical journals ICMJE/2006.

### 2.2. Animal Feeding and Conditions

A total of 12 Anglo-Arabian foals were enrolled in the trial. All animals were properties of the Autonomous Region of Sardinia (RAS), born and raised at the department of Research for Equine Breeding and Production (DREBP) of Agris (Agency of Research of the RAS). In the department, all mares underwent artificial insemination and foals enrolled were all born within one week of the first, in the month of April, in spring at the boreal hemisphere. 

The totality of suckling foals spent the first six months of life with the mare. Foals with mare grazed together on one same pasture (31 hectares), within the limits of DREBP field properties, from April to October. The foals’ diet was based essentially on mare’s milk. Fresh fodder in late spring or home-grown hay (oat, ryegrass and clover-based hay) in summer was offered with concentrate feeds to lactating mares. Throughout the suckling period, all foals were kept on pasture during daytime and sheltered in individual boxes with the mare at night.

Abrupt weaning was carried out when foals got 25–26 weeks old (at 6 mo. ±3 days of age), in the month of October. Each foal was weighed on a digital scale (max. weight 800 kg) housed in an individual box for one month and fed on hay and compound feed (Table 1) *ad libitum.* Free access to water was always available. Straw litter was maintained in the best condition. Feed provision exceeded the amount calculated at 2.7% of body weight (BW), as the capacity of dry matter intake per day (DMI) across all stages, based on consumption observed in previous trials [5]. 

Weaned foals were sent to pasture when 29–30 weeks old (at 7 mo. ±3 days of age) and were supplemented with concentrate feed when in the box, offered to assure a daily leftover weighing not less than 100 g. Hay was always available. Such a feeding regime was maintained until foals reached one year old. 

In spring, foals spent most of the daytime hours on pasture and were sheltered in box stalls during the nighttime. Partitioning of pastures distinguished sewed vs. spontaneous botanical composition. Mostly, ryegrass and clover pastures are used in alternation with spontaneous vegetation. In the box, hay was always available. During summer, foals were fed on hay and compound feed. 

Each foal in an individual box received hay *ad libitum*. Daily administration of hay was scheduled at 6:00 a.m. and at 2:00 p.m. Concentrate feed was based on a mixture between pelleted feed and oat grains (50:50). Daily administration of concentrate feed was scheduled at 11:00 a.m. and at 5:00 p.m. The chemical composition of pellet and of oat grain is reported in Table 1. 

Body condition scoring was adapted from Henneke and co-workers [16]. Each foal was evaluated as to fatness condition following “underweight”, “normal weight” or “overweight” assessment (by point scale conversion 1–9: underweight 1–3; normal weight, 4–6; overweight: 7–9 points), at visual inspection and palpation of sub-cutaneous fat depots at the level of the neck, withers, ribs, croup and base of the tail. One experienced technician, whom foals were accustomed to, assessed fatness condition of all foals throughout the ages (6, 12 and 18 months of age) monitored in this investigation. 

In each change of management, all foals were assessed for body weight on a digital scale (max weight 800 kg) at weaning and subsequently after 30, 180 and 365 days from weaning. 

At the age of 12 months (~65% adult body weight), height at wither (WH) was recorded for each foal by means of a measuring stick. At the age of 18 months, ~78% of adult body weight is assumed be achieved and WH was recorded [17].

### 2.3. Blood Analysis

All animals underwent physical examination by the veterinary surgeons of the DREBP. Foals were destined for sport activities and health maintenance with best practice for expression of athletic potential is the goal of the experimental stable. 

During the physical examination of foals, individual whole blood sampling was carried out following DREBP internal protocols and two aliquots were used for the purposes of this investigation. Blood sampling was scheduled at 8:00 a.m., 2 h after hay administration and 3 h prior to concentrate feeding. Blood was drawn into vacuum tubes directly, through the puncture (18-gauge needle) of the jugular vein. Samples were kept refrigerated by storing tubes in polystyrene cases in the upright position in a cooling bag, to ensure adequate temperature during sample transfer to the laboratory. All samples were identified with foal’s name, electronic individual code (EIC) and date of sampling and processed within 6 h from collection for complete blood count (CBC). On whole blood stored in ethylenediaminetetracetic acid (K_2_-EDTA) containing tubes, parameters were quantified through an automatic analyzer (Mindray BC-5000 Vet, Alcyon, Italy). 

Individual serum was screened for complete biochemical profile. Samples underwent centrifugation at 1500× *g* for 10 min. The serum was removed and stored in a vial (2 mL) to be frozen at −20 °C until analysis. All samples were analyzed within one week, through an automatic biochemical analyzer (Mindray BS-200, Alcyon, Italy) for the determination of serum concentration of ubiquitous intermediate metabolites (including urea, non-esterified fatty acids, NEFA; beta-hydroxy-butyrate BHBa), hepatic enzymes (alanine transaminase, ALT; aspartate aminotransferase, AST; gamma-glutamyl transferase, γ-GT) and other biochemical parameters (total bilirubine, TBil; total protein, TP; total cholesterol, TC; total triglycerides, TG; glucose, GLU; lactate dehydrogenase, LDH; creatine phosphokinase, CPK; alkaline phosphatase, ALP; calcium, Ca; phosphorus, P; iron, Fe). Reference intervals were adjusted for horse species and developed internally for the foal. Additionally, biochemical parameters obtained were compared to those reported by Kaneko and co-authors [18] and Latimer and co-authors [19].

### 2.4. Analysis of Data and Statistics

Body weight (BW) was recorded for each foal, and differences between the final BW and initial BW of each stage was calculated. Average daily gain (ADG) was therefore calculated for weanlings and yearlings. ADG was used for the prediction of daily energy requirements. Daily energy requirement, expressed as digestible energy (DE) was predicted with the formula [20]:

(1) $$\mathrm{DE}\;\left(\mathrm{Mcal}\times d^{-1}\right)=\left\{\left[\left(56.5x^{-0.145}\right)\times \frac{\mathrm{BW}}{1000}\right]+\left[\left(1.99+1.21\times x^{2}\right)\times \mathrm{ADG}\right]\right\}$$

where x is the age of foals in month of the foal and ADG is average daily gain in kg per day. 

The chemical composition of hay and compound feeds regimes was used to estimate the digestible energy (DE) content per kg dry matter (DM) in feed. Calculations were based on the following formula [21]:

Concentrates:

(2) DE (Mcal/kg DM)=4.07−0.055 (%ADF)

Forages:

(3) $$\begin{array}{c} \mathrm{DE}\;(\mathrm{Mcal}/\mathrm{kg}\;\mathrm{DM})=2.118+0.01218\mathrm{CP}-0.00937\mathrm{ADF}-0.0383\left(\mathrm{NDF}-\mathrm{ADF}\right)+0.04718\mathrm{EE}\;+\\ 0.02035\mathrm{NFC}-0.0262\mathrm{Ash}, \end{array}$$

The statistical analysis of data was carried out by using the ANOVA in a mixed procedure model:

Y_i,j,k,l_ = μ + D_i,j_ + G_k,l_ +H + e_i,j,i,j,k_ (4)

where Y is the dependent variable (CBC parameters; biochemical parameters), μ is the overall mean, D is the fixed effect of the sampling time (three levels: 6 mo.- vs. 12 mo.- vs. 18 mo.-old), G as covariate for fatness (1 = underweight; 2 = normal weight; 3 = overweight). The animal (H) was the random effect, and e the random error.

Confidence intervals and grouping were adjusted according to Tukey’s method. All data were analyzed using SAS 9.2 (SAS Inst. Inc. Cary, NC, USA). Statistical significance was set for *p* < 0.05, whereas *p* < 0.10 represented a trend.

Selected parameters with biological significance for the clinical evaluation of the metabolic condition were also processed at Pearson’s test for the assessment of potential correlation (ρ < 0.300 = weak correlation; 0.300 < ρ < 0.600 = mild correlation; 0.600 < ρ < 1.000 strong correlation; +ρ or –ρ, positively or negatively correlated, respectively; significance for *p* < 0.005).

## 3. Results

All animals enrolled in the trial appeared healthy throughout the experimental period. Hemogram of foals at different growth stages is reported in Table 2.

Despite apparently healthy, yearlings on spring pasture showed a significantly higher value of WBC and NEU according to sampling time. However, such increase displayed to be very limited, if considered in view of the reference range, and not associated with clinical manifestation. A marked animal effect could be pointed out for other parameters of the hemogram, which however fell in the reference range. Worth of note, the high HGB levels (increasing concentrations above the reference range) in association with MCHC near to the upper value of the reference range. The correlation between some parameters of the CBC at Pearson’s test highlights the known species-specific peculiarities of RBC of the horse, also in foals, in comparison with other animal species. Correlation coefficients and significance are reported in Table 3. At the microscopic analysis of blood smears, no rouleaux were pointed out and all samples considered suitable for diagnostic use. 

The markedly positive correlation of RBC count and HBG (ρ = 0.860, *p* < 0.001) could be expected, likewise the correlation between RBC and HCT (ρ = 0.875, *p* < 0.001). The moderately negative correlation between MCV and RBC count (ρ = −0.605, *p* = 0.028) can be considered a peculiarity of this species and has no prognostic value.

The metabolic profile of foals at different growth stages is reported in Table 4.

The metabolic profile varied according to sampling time for several parameters. Marked individual effect could be also found for some metabolites. In particular, UREA levels differed significantly (*p* = 0.006) and exceeded the upper value of the reference range in 12-mo. old foals. Additionally, ALT significantly increased (*p* = 0.008) in 12-mo. old foals but levels were always within the reference range and this datum was not of prognostic value. AST showed a similar trend, yet being significant for sampling time, meanwhile for individual foal (*p* = 0.023) (Table 4).

Circulating total calcium showed a significant decrease (*p* = 0.029) from 6-month to 18-month-old foals, still being always within the physiological range. Similarly, sideremia decreased significantly (*p* < 0.0001) with age of foals, without affecting the HGB level.

NEFA levels showed a peak in 12-month-old foals and markedly decreased when foals were 18 months old (*p* = 0.001). Concomitantly, BHBa synthesis occurred at NEFA decrease, being however of no prognostic value.

Correlation coefficient between selected biochemical parameters involved in energy balance exploration are reposted in the Table 5. Marked correlation (ρ = 0.738) were found highly significant (*p* < 0.001) between circulating UREA and ALT. In addition, UREA levels were significantly found (*p* = 0.033) moderately correlated (ρ = 0.503) with AST levels. In addition, circulating ALT and AST were significantly (*p* = 0.11) moderately correlated (ρ = 0.538).

Worth of note, γ-GT (enzyme of liver tissue damage) turned out to be significantly and positively correlated with AST (ρ = 0.577; *p* = 0.012) and very close to the significance with the increase of BHBa synthesis (ρ = 0.467; *p* = 0.051). However, γ-GT levels always fell within the physiological range for the horse over time. 

UREA and ALT levels increase in 12- mo. old foals to decrease slowly in 18-mo. old foals. Meanwhile, AST displays to increase non significantly in foals up to 18 mo. of age, when also BHBa synthesis increase significantly (*p* = 0.006). All liver enzymes (ALT, AST and γ-GT) turned out to be within the reference range throughout the trial. Only UREA increased (43.7 ± 8 mg/dL) above the upper limit of the reference range (26–41 mg/dL). 

Feed and energy intake from data in stable and digestible energy intake estimation as well as percentage of intake to cover energy requirements are reported in Table 6. All foals appeared constantly lean, though judged within the range of normal weight, as estimated in this trial.

## 4. Discussion

The metabolic profile for monitoring the evolution of systemic energy balance from developing tissues displayed to be useful for the purposes of this trial. Very promisingly, in fact, the selected metabolites analyzed in this trial were able to provide a picture of circulating energy substrates, across the different growth stages of the foal, from weaning to the age of 18 months. The response of the foal to feeding and management practices shows the complexity of interactions between internal factors (conversion of energy from the diet or body storage re-directed to developing tissues) and environmental factors (diet, management, season). Such interactions lead to different metabolic patterns and energy balance rates across growth stages, over time.

The analysis of selected biochemical parameters explored in this trial allows us to draw a series of considerations. 

One first aspect deals with the importance of screening the different growth stages in view of the changes of feeding management. Here, we screened the effect of consolidated feeding and management practices, developed within the DREBP, oriented to the production of sport horses. However, several aspects from field evidence can now be supported by laboratory data. 

At Mediterranean latitudes, in view of the favorable climate condition, the practice to send foals to pasture after weaning is common. When out to the field, the foal is allowed to express a broader range of movements that requires the coordination of muscular activity. This could be of help for adequate muscular development and to improve the robustness of periostal tissue, above all, favoring the natural behavior (including grazing) of the foal and stimulating the socialization [21,22] 

Focusing on energy balance across growth stages, the increase in circulating NEFA and UREA above the upper limit of the reference range in 12-month-old foals could be expected. When foals were allowed to graze on pasture, it could be argued that energy intake could not meet daily energy requirements. In fact, despite more than the 50% of energy came from compound feed consumed when the foal stayed in the box (see Table 6), it is arguable that while in the field the foal did not spend the time grazing as an adult horse would. In doing so, a decrease of DM intake along with higher energy expenditure could be expected. However, all the foals demonstrated expected ADG and growth rates if compared to previous investigations [5]. Our results are in agreement with data reported in the literature by other authors [7]. 

Despite the fact that a negative energy balance could be observed, the involvement of the liver was not clinically significant and levels of enzymes to assess liver failure turned out to be of no prognostic value. In addition, selected enzymes for the evaluation of muscular fatigue (CK and LDH) turned out to be constantly within the reference range across the different growth stages, over time. 

The different substrates used for energy production are worthy of being commented on in detail.

The use of nutrients and substrates can give rise to different metabolic pathways that are largely dependent on species diversity and successful adaptation to the natural diet. Thus, according to energy levels required during growth, the change of the diet across the different growth stages explored in this trial would have reasonably affected the utilization and distribution of nutrients. 

The horse, as a monogastric herbivore, demonstrates to be well adapted to high forage proportion in the diet [23]. Efficient hindgut fermenter, the horse shows good digestibility rates of non-fibrous macronutrients in the foregut as well [24]. The majority of fiber fermentation occurs in the hindgut thanks to the metabolic activity of microbial populations. End products (volatile fatty acids VFA, namely acetic, butyric and propionic acid) from microbial fermentations represent important fuel sources for the horse. In particular, propionic acid represents the carbon (C3) backbone for gluconeogenesis, meanwhile acetic acid (C2) is the main substrate for peripheral tissues and stored as fats in the case of positive energy balance. Butyric acid is converted to β-hydrohybutyrate in the liver as an energy source [25]. Thus, the change of the diet composition could lead to a shift in the utilization of energy from nutrients. In general, the horse can digest efficiently both NSC as well as fibrous carbohydrates, thus glucose, as well as VFA, can be used for energy purposes and, if in excess, stored in tissues [15]. However, also amino acids can be used by the horse for energy purposes. The deamination of amino acids leads to the increase of ammonia that is converted by the liver into UREA, to ease the clearance [25]. The significant correlation between UREA and both AST and ALT can witness the role of the liver in the conversion of ammonia generated by amino acid deamination for energy purposes, which is arguable if low energy intake is observed, as in this case. Fat mobilization from body depots is witnessed by the marked increase of NEFA in the bloodstream of foals aged 12 mo. This occurrence is concomitant to the UREA increase, which allows us to consider that NEFA and amino acids were used as a substrate for energy production. 

In this trial, UREA and NEFA decreased in foals of 18 months, β-HBa acid increased significantly, being below the limit of detection in 6-month-old foals. Independent of sampling time, BHBa turned out to be below the lower limit of the reference range for the horse. It is to point out, however, that the range is referred to adult horses. To the best of our knowledge, no other reference range are available in the literature at present. In any case, the increase of BHBa may be symptomatic of an alternative energy source with circulating NEFA reduction, likely due to reduction of fat depots to redirect energy to other developing tissues, such as muscle masses, being muscle tissue a high consumer of fatty acids. Noteworthy, the correlation between γ-GT and UREA, AST and BHBa (the latter very close to significance, *p* = 0.051) points to a certain involvement of liver function. Recently, Newman and Verdin [26] pointed to the epigenetic effects of BHBa in determining energy partitioning in different cells of the organism, in view of other sites than the liver capable of synthesizing BHBa from fatty acids for energy purposes (kidney and intestine). Consistently, γ-GT always dropped within the physiological range.

Finally, the marked decrease of Ca and Fe levels, reaching the lowest value in 18-month-old foals, should be interpreted in light of the development of tissues over time. The skeleton and muscle tissues start to become more robust and increase in mass, respectively. In fact, in the growing horse Ca is involved in the ossification of bones. Fe is involved in myoglobin synthesis and probably those levels are an expression of what happens in 18-month-old foals.

## 5. Conclusions

The analysis of energy balance in foals demonstrates the importance of continuous monitoring of animals throughout the growth stages. The development of tissues requires the utilization of different substrates, thus providing indications about sources and distribution within the animal body. That way, nutritional and feeding plans may be adjusted in the case of clinical manifestation or to prevent the onset of metabolic disorders during growth.

## Figures and Tables

**Table 1:** Rations for the growing horse across different growth stages.

Item ^2^	Age ^1^		
	6 mo.	12 mo.	18 mo.
Ingredient (kg/d per horse, as fed)			
Hay ^3^	4.00	5.00	7.00
Compound feed	3.00	4.00	4.00
Pasture	−	+	+
Chemical composition of compound feed (g/kg feed)			
DM	881		
NDF	553		
CP	140		
Ash	135		
Ether extract	40.5		
DE (Mcal/kg DM) ^4^	3.32		

^1^ Age: 6 mo., foal aged six months at weaning; 12 mo., foal at one year; 18 mo., foal aged 18 months. ^2^ Item: DM, dry matter; NDF, neutral detergent fiber; CP, crude protein; ^3^ Hay (oat/ryegrass/clover based) chemical composition: DM = 84.7%; CP = 12.9% of DM; NDF = 58.5% DM; ADF = 34.2% of DM; Ash = 13.5% of DM; Ether extract = 2.16% of DM; DE (Mcal/kg DM) = 2.44. ^4^ DE = digestible energy per kg of dry matter in feed expressed as mega calories. Energy density for concentrate and forage was predicted by the formula of NRC, 2007.

**Table 2:** Hemogram (CBC) of foals across the different growth stages.

Item ^2^	Reference Range ^3^	Age ^1^				*p*-Value ^4^	
		6-mo.	12-mo.	18-mo.	Pooled-SD	Sampling Time	Animal
WBC (10^9^/L)	4.90–10.3	10.3	12.7	11.1	2.051	0.175	0.024
LYMPH (10^9^/L)	2.20–8.10	5.31	4.37	4.73	1.056	0.425	0.004
NEU (10^9^/L)	1.70–5.80	4.45	6.71	5.03	1.093	0.010	0.028
MONO (10^9^/L)	0.00–1.52	0.39	0.44	0.42	0.108	0.494	0.115
EOS (10^9^/L)	0.00–0.80	0.12	0.27	0.19	0.158	0.179	0.491
BASO (10^9^/L)	0.00–0.30	0.05	0.04	0.04	0.022	0.443	0.889
RBC (10^12^/L)	6.20–10.2	8.13	8.25	8.38	0.956	0.909	0.005
HGB (g/L)	63.0–132	132	139	141	22.06	0.514	0.255
HCT (%)	31.0–50.0	34.0	36.1	36.3	2.293	0.255	0.016
MCV (fL)	37.0–53.0	41.0	40.9	43.5	1.852	0.062	<0.0001
MCH (pg)	14.0–20.0	15.8	16.5	16.9	0.741	0.239	0.854
MCHC (g/L)	360–390	386	388	389	36.75	0.891	0.775
PLT (10^9^/L)	127–206	122	125	139	24.54	0.608	0.454
PCT (ML/L)	0.70–2.10	0.79	0.88	0.85	0.170	0.408	0.280

^1^ Age: 6-mo. = weanling at 6 months of age; 12-mo. = yearling at 12-months of age; 18-mo. = yearling at 18-months of age. ^2^ Item: WBC = white blood cell; LYMPH = lymphocyte; NEU = neutrophil granulocytes; MON = monocytes; EOS = eosinophil granulocytes; BASO = basophil granulocytes; RBC = red blood cell; HGB = hemoglobin; HCT = hematocrit; MCV = mean cell volume; MCH = mean cell hemoglobin; MCHC = mean cell hemoglobin concentration; PLT = platelet count; PTC = platelet-crit. ^3^ Reference value according to Kaneko et al., 2008 and Latimer et al., 2011. ^4^ *p*-Value: *p* < 0.05 indicates significant effect of linear and/or quadratic contrasts.

**Table 3:** Correlation coefficients (ρ) and significance (*p*-value in italics below ρ) between selected parameters of CBC at Pearson’s test.

Item ^1^	HGB	RBC	HCT	MCHC	MCV
RBC	0.860				
	*0.000*				
HCT	0.854	0.875			
	*0.000*	*0.000*			
MCHC	0.889	0.620	0.525		
	*0.000*	*0.024*	*0.065*		
MCV	−0.360	−0.605	−0.146	−0.416	
	*0.226*	*0.028*	*0.635*	*0.157*	
WBC	0.530	0.373	0.689	0.300	0.333
	*0.063*	*0.210*	*0.009*	*0.319*	*0.266*

^1^ Item: WBC = white blood cell; RBC = red blood cell; HGB = hemoglobin; HCT = hematocrit; MCV = mean cell volume; MCH = mean cell hemoglobin; MCHC = mean cell hemoglobin concentration.

**Table 4:** Metabolic profile of foals across the different growth stages.

Item ^2^	Reference Range ^3^	Age ^1^				*p*-Value ^5^		
		6-mo.	12-mo.	18-mo.	Pooled-SD	Sampling Time	Fatness	Animal
TP (g/L)	52.0–79.0	73.1	76.1	73.2	10.9	0.744	0.854	0.014
UREA (mg/dL)	26.0–41.0	40.1	43.7	32.1	8.137	0.006	0.454	0.337
ALT (U/L)	10.0–24.0	8.27	14.3	10.7	4.112	0.008	0.246	0.353
AST (U/L)	226–366	266	307	312	67.05	0.260	0.802	0.029
γ-GT (U/L)	10.0–32.0	17.3	14.7	19.5	5.361	0.127	0.725	0.200
TB (mg/dL)	0.00–3.20	1.73	1.32	1.41	0.584	0.266	0.687	0.118
Ca (mmol/L)	2.80–3.40	3.47	3.22	2.92	0.440	0.029	0.445	0.179
P (mmol/L)	1.00–1.18	1.81	1.78	1.73	0.310	0.848	0.998	0.112
Fe (μmol/L)	13.0–37.0	37.7	32.3	12.1	10.52	<0.001	0.025	0.907
CK (U/L)	190–370	334	493	391	183.5	0.150	0.142	0.359
LDH (U/L)	112–456	242	258	291	86.62	0.365	0.316	0.121
Glu (mmol/L)	3.50–5.90	8.47	6.66	4.72	1.781	<0.001	0.020	0.224
TG (mmol/L)	0.26–2.60	0.31	0.29	0.29	0.13	0.866	0.959	0.001
NEFA (mmol/L) ^4^	0.02–0.43	0.18	0.86	0.25	0.283	0.001	0.440	0.906
BHBa (mg/dL) ^4^	1.00–3.10	0.00	0.00	0.05	0.003	0.006	0.214	0.690

^1^ Age: 6-mo. = weanling at 6 months of age; 12-mo. = yearling at 12-months of age; 18-mo. = yearling at 18-months of age. ^2^ Item: TP = total protein; ALT = alanine aminotransferase; AST = aspartate aminotransferase; γ-GT = gamma alkaline phosphatase; TB = total bilirubin; CK = creatine kinase; LDH = Lactic dehydrogenase; GLU = glucose; TG = total triglycerides; NEFA = non-esteriefied fatty acids; BHBa = beta-hydroxy-butyric acid. ^3^ Reference value: according to Kaneko et al., 2008 and Latimer et al., 2011 for adult horses. ^4^ Reference value: Cornell University College of Veterinary Medicine, Animal health diagnostic center. ^5^ *p*-value: *p* < 0.05 indicates significant effect of linear and/or quadratic contrasts.

**Table 5:** Correlation coefficient (ρ) and significance (*p*-value, in italics) at Pearson test between NEFA, BHBa, UREA and hepatic enzymes.

Item ^1^	NEFA	γ-GT	AST	BHB	ALT
γ-GT	−0.144				
	*0.567*				
AST	−0.130	0.577			
	*0.607*	*0.012*			
BHBa	−0.202	0.467	0.269		
	*0.422*	*0.051*	*0.281*		
ALT	0.297	0.282	0.582	−0.239	
	*0.232*	*0.258*	*0.011*	*0.339*	
UREA	0.366	0.080	0.503	−0.346	0.738
	*0.136*	*0.753*	*0.033*	*0.159*	*0.000*

^1^ Item: γ-GT = gamma-glutamil transferase; ALT = alanine aminotransferase; AST = Aspartate aminotransferase; BHBa = beta-hydroxy-butyrate acid; NEFA = non-esterified fatty acids; ALT = alanine aminotransferase.

**Table 6:** Body weight (BW, kg), average daily gain (ADG, kg/d) and intake of energy from compound feed in foals at different growth stages.

Item ^2^	Age ^1^		
	6-mo.	12-mo.	18-mo.
Foal BW (kg)	194 ± 15.6	327 ± 23.6	402 ± 27.2
ADG (kg/d)	0.76 ± 0.12	0.66 ± 0.08	0.46 ± 0.04
Daily DE intake (Mcal)	7.82 ± 0.21	11.6 ± 0.40	12.0 ± 0.18
% of DE daily requirement ^3^	52.3 ± 1.40	53.4 ± 1.83	52.0 ± 0.78

^1^ Age: 6-mo. = weanling at 6 months of age; 12-mo. = yearling at 12-months of age; 18-mo. = yearling at 18-months of age. ^2^ Item: BW = body weight; ADG = average daily gain (kg/d); DE = digestible energy. ^3^ Percentage of daily DE requirement predicted from compound feed intake per day.

## Data Availability

Not applicable.
